# Iatrogenic Ureteropelvic Junction Disruption from Lumbar Spinal Fusion Surgery: Early Repair using The SP Robotic System

**DOI:** 10.1590/S1677-5538.IBJU.2024.0690

**Published:** 2025-01-23

**Authors:** Arianna Biasatti, Leslie C. Licari, Eugenio Bologna, Angelo Orsini, Matthew C. Pearson, Riccardo Autorino

**Affiliations:** 1 Rush University Medical Center Department of Urology Chicago IL USA Department of Urology, Rush University Medical Center, Chicago, IL, USA

## Abstract

**Introduction:**

Severe iatrogenic ureteral injuries are uncommon but challenging clinical scenarios, mostly related to abdominal and gynecological surgery (1) but lately also to spinal surgery (2). Prompt management is mandatory to avoid impaired outcomes (3). Moreover, a minimally invasive approach is desirable to minimize surgical morbidity and expedite recovery (4). Single Port robotic surgery is being implemented for a variety of indications, including ureteral surgery (5, 6).

**Materials and Methods:**

We present the case of a 48-year-old, who underwent lumbar spinal fusion surgery and 3 days after discharge was readmitted presenting abdominal pain. A CT scan revealed a large abdominal fluid collection and consequently a percutaneous drain was placed. A subsequent CT-urogram revealed a right ureteral injury at level of the ureteropelvic junction (UPJ). A percutaneous nephrostomy was inserted after unsuccessful retrograde and anterograde stent placement attempts. The patient underwent SP robotic early repair of the ureter 3 weeks after spinal surgery.

**Results:**

SP robotic ureteral injury repair with transperitoneal approach was performed. The surgery was well tolerated without intraoperative complications, patient was discharged on post-operative day 2. Right percutaneous nephrostomy was removed after 2 weeks and ureteral stent after 4. At 6-months follow-up the patient was asymptomatic and CT-urogram confirmed symmetric contrast excretion without hydroureteronephrosis or contrast leakage.

**Conclusion:**

SP robotic repair of the UPJ injury is safe and feasible. This procedure provides the benefits of minimally invasive surgery, and it should be considered as a valid alternative to traditional multiport robotic approach.

Available at: http://www.intbrazjurol.com.br/video-section/20240690_Autorino_et_al
